# Timing of tumor-induced atelectasis resolution and pulmonary function restoration in the course of image-guided moderate hypofractionated thoracic irradiation: a case report and mini-review of literature

**DOI:** 10.1259/bjrcr.20200168

**Published:** 2021-10-18

**Authors:** Raphael Bodensohn, Lukas Käsmann, Chukwuka Eze, Montserrat Pazos, Claus Belka, Farkhad Manapov

**Affiliations:** 1 Department of Radiation Oncology, University Hospital, LMU Munich, Marchioninistraße 15, 81377 Munich, Germany; 2 German Cancer Consortium (DKTK), Munich, Germany

## Abstract

This case report describes a patient with squamous cell carcinoma of the lung (cT4 (Infiltration of left pulmonary artery) cN2 cM0, TNM eighth edition) and subsequent tumor-induced atelectasis of the left upper lobe. Despite initially presenting himself with a poor performance status (ECOG-PS III) and diminished lung function, the patient was treated with image-guided thoracic irradiation to a total dose of 45.0 Gy (to the whole planning target volume) / 52.5 Gy (as simultaneous integrated boost to the Primary Tumor) applied in 15 daily fractions. Through the radiation treatment, the upper lobe could be reaerated, and the patient’s lung function and performance were improved.

## Clinical case

**Figure 1.**
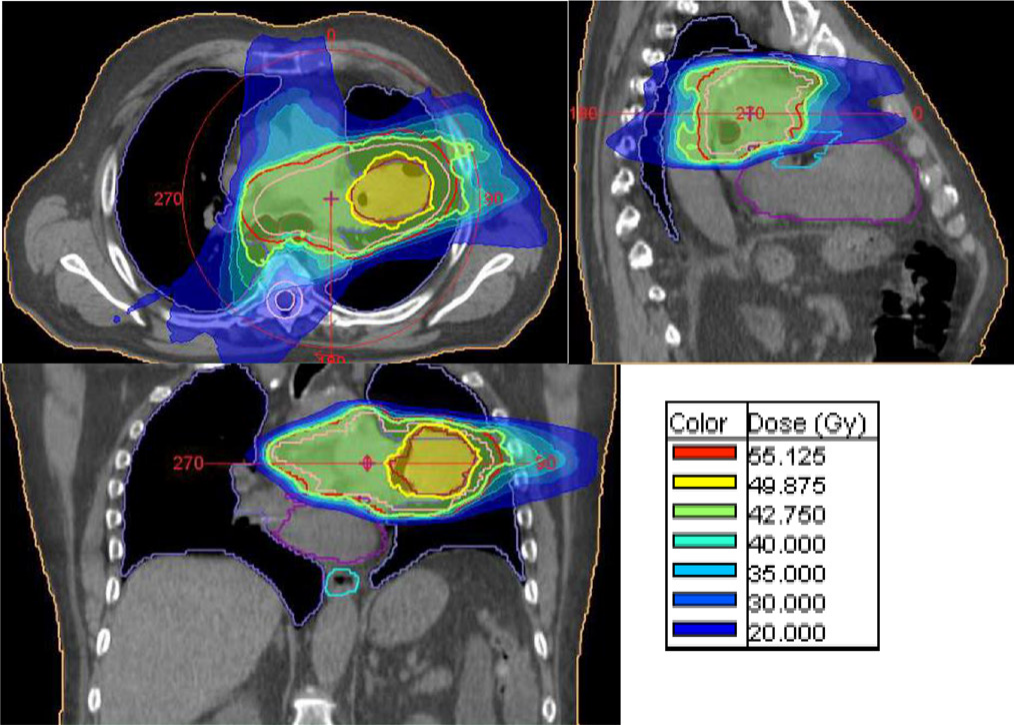
Images depicting the radiation plan used for treatment. Atelectasis occurred between planning-CT and start of irradiation.

A 74-year-old male patient with a squamous cell carcinoma of the lung cT4 cN2 cM0 (TNM eighth edition) obstructing the left upper bronchus presented himself to our clinic with severe dyspnea (Modified Medical Research Council Dyspnea Scale Grade III)^1^ and diminished baseline pulmonary function as follows: FEV1 = 1.43l (48.1% predicted) and VCmax = 1.82l (44.0% predicted). Definitive treatment, *i.e*. surgery or conventional chemoradiation, was not feasible due to the patient’s general condition (ECOG-PS III) and limited lung function. In order to relieve symptoms and halt local tumor progression, the involved lymph node stations and primary tumor mass were treated in volumetric modulated arc therapy technique with 3.0/3.5 Gy per fraction to a total dose of 45.0/52.5 Gy (EQD2/BED = 48.8/58.5 Gy and 59.1/70.9 Gy, assuming α/β = 10 for the tumor), respectively. Planning-CT was performed conventionally; no additional imaging was used. Dose constraints were as following: total lung V20 <20%, total lung mean dose <10 Gy, esophagus V30 <25%, heart mean dose <10 Gy, spinal cord maximal dose <30 Gy. The dose constraints are based on previously published works on image-guided moderate hypofractioned thoracic irradiation.^2,3^ No CTV was defined due to the diminished lung function; gross tumor volume to planning target volume expansion was 9 mm craniocaudal and 6 mm axial. The irradiation plan is depicted in Figure 1.

**Figure 2.**
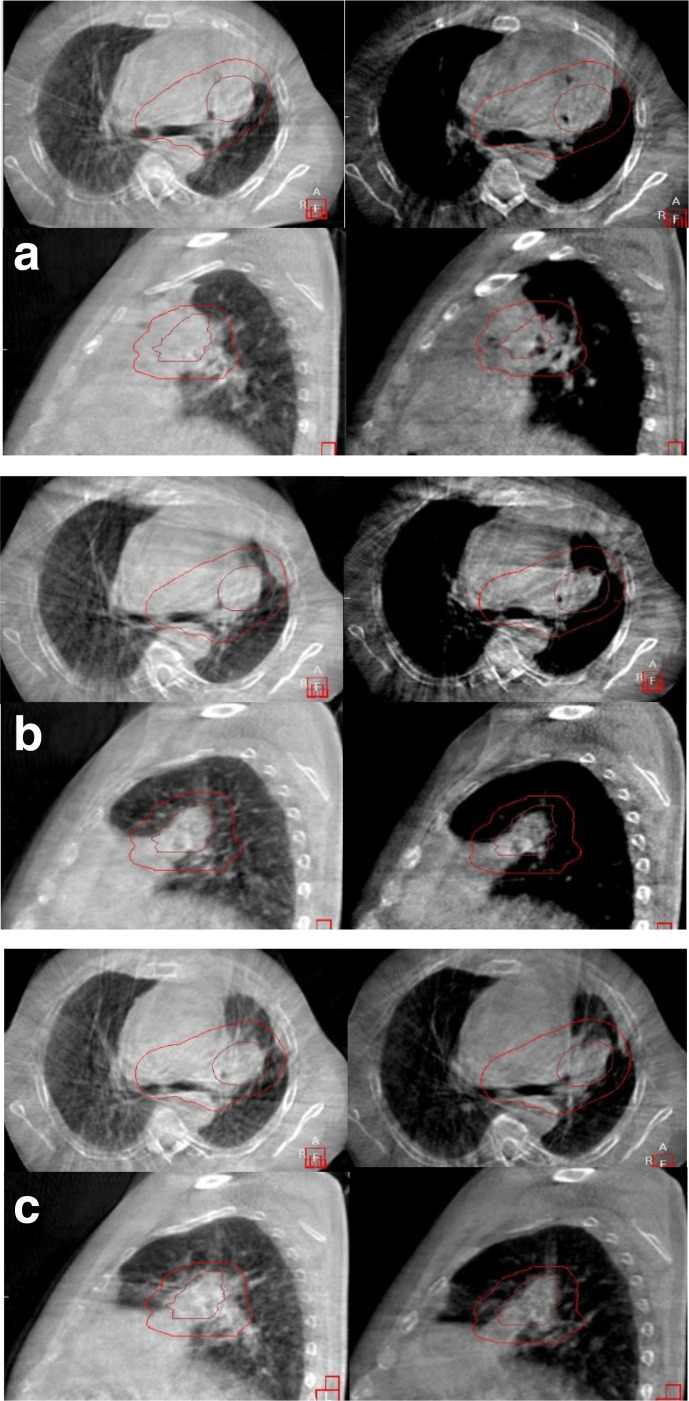
Development of the atelectasis during thoracic irradiation. (A) End of 04/2020: first day of irradiation – atelectasis of the left upper lobe can be seen on kilovoltage CBCT. (3.5/52.5 Gy; EQD2 (α/β 10): 3.9 Gy). (B) Early 05/2020: reaeration of the left upper lobe bronchus begins. Segmental bronchi are opening. The patient reports improvement in dyspnea (45.5/52.5 Gy; EQD2 (α/β 10): 51.19 Gy). (C) Mid 05/2020: on CBCT before the last fraction, almost complete resolution (52.5/52.5 Gy; EQD2 (α/β 10): 59.06 Gy). Lung function examination: 03/2020 Prior to Radiotherapy: FEV1 1.43l (48.1% predicted), VCmax 1.82l (44.0% predicted) Early 06/2020 After Radiotherapy FEV1 2.49l (82.6% predicted), VCmax 3.17l (76.6% predicted). CBCT, cone-beam CT.

Image guidance was performed with kilovoltage cone-beam CT prior to each fraction to monitor possible interfractional changes of the tumor volume or position.^4^ During the course of treatment and following 11 fractions with a cumulative dose to the tumor of 38.5 Gy (EQD2/BED = 43.3/52.0 Gy), accentuated segmental bronchi and beginning inflation of the left upper lobe was observed on the cone-beam CT. Symptomatically, improvement in dyspnea from Grade III to Grade II occurred. After 13 fractions (45.5 Gy; EQD2/BED = 51.2/61.4 Gy), inflation of the involved lobe was more pronounced, and on completion of radiotherapy, the atelectasis had almost completely resolved. Due to no significant change of the target volume in relation to the organs at risk despite of the reaeration, no adaption was necessary. Furthermore, improvement of performance status (ECOG II) and pulmonary function (FEV1 = 2.49l (82.6% predicted), VCmax = 3.17l (76.6% predicted)) was observed. The reaeration of atelectasis in the course of image-guided moderate-hypofractionated thoracic irradiation is depicted in Figure 2.

## Review of literature

In 1986, Majid et al emphasized the importance of thoracic irradiation in the management of tumor-induced atelectasis.^5^ Though rather rare, radiation treatment can be very effective in restoring patients’ pulmonary function. In a study by Clarke et al, change of atelectasis was observed in 9% of cases.^6^ However, timing (*i.e.* time interval from atelectasis detection to the start of thoracic irradiation) seems to be pertinent for successful treatment: Reddy et al identified in an observatory study that re-expansion of the atelectatic area was achieved in 71% of the patients who were irradiated within 2 weeks after atelectasis occurred, whereas this was the case in only 23% of the patients who were irradiated 2 or more weeks after.^7^ As tumor-associated atelectasis was diagnosed just before start of radiotherapy, timing seems to have been convenient for reaeration to occur. Delivery of a total dose of 51.5/3.5 Gy to the obstructing primary tumor also was sufficient. According to Chetty et al, reaeration of tumor-obstructing bronchial stems was achieved significantly more often if radiation doses of 50.0 Gy or higher in 2.0 Gy fraction doses were administered.^8^ An overview of the literature is given in Table 1.

**Table 1.** Literature overview

Study	Total number of patients, and patients with atelectasis	Total dose in Gy (1.8–2.0 Gy SF)	Number of patients with improvement of atectasis after radiation therapy	Special feature
Majid et al. 1986	*N* = 33; 28 patients atelectasis and NSCLC	12.0–60.0	17/28 (61%)	9/13 (80%) with doses of 50.0 Gy recovered
Clarke et al. 2019	*N* = 430; 48 patients with pre-existing atelectasis	50.0–60.0	10/48 (21%)	20 patients developed new or progressive metastases
Reddy et al. 1990	*N* = 22; 12 patients with atelectasis	54.0–70.0	10/12 (83%)	Changes in atelctasis were associated with displacement of primary tumor
Chetty et al. 1989	*N* = 57; all patients had atelectasis	30.0–60.0	12/57 (21%); 3/57 (5%) partial response; 9/57 (16%) complete response	All patients who responded received more than 50.0 Gy

NSCLC, non-small cell lung cancer.

## Discussion

This case shows a successful resolution of a tumor-induced atelectasis through image-guided moderate hypofractionated thoracic irradiation. Initially, the patient’s dyspnea and performance status might have discouraged more aggressive treatment. However, individually accelerated hypofractionated radiotherapy led to swift palliation. In parallel, significant improvement of lung function could directly be seen in the change of FEV1 [1.43l (48.1% predicted) before and 2.49l (82.6% predicted) after treatment] and VCmax [1.82l (44.0% predicted) before and 3.17l (76.6% predicted) after treatment]. Unfortunately, patients’ initial general physical condition is often the reason for withholding needed intensive treatment. In the case of deterioration due to disease itself, it is important for the managing physician to closely evaluate if treatment might actually ameliorate patients’ condition. Furthermore, it is essential that treatment is not delayed and the sufficient total radiation dose is used. The literature review showed that patients’ condition improved most if at least 50.0 Gy in 2.0 Gy daily fractions was applied. Another important component of successful thoracic irradiation is daily image guidance with kilovoltage cone-beam CT, as the target volume can substantially change between treatment fractions; adaptive treatment may also be discussed for some patients.^4^ In the above case, plan adaptation was not necessary, but one should be wary of cases which might require replanning.^9^ With the emergence of adaptive radiotherapy units including MR-LINAC, on-table adaptation will be easier to achieve.^10^

## Conclusion

In summary, this case demonstrates that it is possible to irradiate patients with a large tumor and tumor-related atelectasis, despite poor initial lung function and performance status and thus alleviate dyspnea, resolve atelectasis and achieve tumor control.

## Learning points

- Poor performance and diminished lung function parameters do not necessarily prevent patients from receiving sufficient treatment.
- Daily image guidance is important for successful treatment visualization and adaptation.
- Timing of treatment initiation and prescription/application of sufficient total radiation dose are important for successful treatment.
